# Diagnostic Pain: A Case of Pudendal Neuralgia

**DOI:** 10.7759/cureus.68096

**Published:** 2024-08-29

**Authors:** Keshav Khanijow, M. Carolina Musri, Matthew Kelly

**Affiliations:** 1 Division of Hospital Medicine, Johns Hopkins Bayview Medical Center, Johns Hopkins University School of Medicine, Baltimore, USA

**Keywords:** rare disease burden, pelvic anal pain, difficult diagnosis, pudendal neuralgia, chronic pain management

## Abstract

Pudendal neuralgia remains a challenging diagnosis given the absence of sensitive biomarkers or imaging findings. The following case describes a patient with rectal pain complicated by prolonged hospitalization who was eventually diagnosed with pudendal neuralgia per the Nantes criteria. It furthermore underscores several confounders that prevented timely diagnosis, including misattribution of her symptoms to prior resolved conditions, anchoring bias in the absence of confirmatory evidence, and misattribution of her pain to opiate-induced allodynia. It also draws attention to the toll exacted by delays in diagnosis, including patient discomfort, deconditioning, exposure to high-dose opioids, susceptibility to potential nosocomial infections, strain on patient-provider relationships, and the need for post-discharge inpatient rehabilitation, in addition to significant financial costs.

## Introduction

Pudendal neuralgia is estimated to affect 1 person per 100,000 and remains a challenging diagnosis [1]. It is thought to be caused by trauma, injury, or inflammation of the pudendal nerve, resulting in extreme pelvic and anal pain [1]. The pudendal nerve arises from sacral spinal nerves S2-S4 and innervates the rectal canal, anus, perineum, and external genitalia with both motor and sensory fibers [2]. Although the initial cause of pudendal neuralgia may not be apparent, there are reports of this happening post-procedurally or due to entrapment of the nerve between ligaments or muscles [2]. Unfortunately, there are no sensitive or specific radiographic signs for pudendal neuralgia, though MRI can potentially rule in the diagnosis if pudendal nerve inflammation or entrapment is identified [1]. The diagnosis for this often remains clinical, especially after multiple negative imaging tests and procedures. To aid in the diagnosis, the Nantes criteria were established. These criteria include (1) pain in the anatomical territory of the pudendal nerve; (2) worsened by sitting; (3) the patient is not woken at night by the pain; (4) no objective sensory loss on clinical examination; and (5) positive anesthetic pudendal nerve block [3]. This case describes a patient with missed opportunities for diagnosis, continued symptomatology with untargeted pain, and consequently prolonged hospitalization.

## Case presentation

A 40-year-old female with a history of controlled ulcerative colitis and systemic lupus erythematosus (SLE) on prednisone and mycophenolate mofetil, hemorrhoidectomy, and fissurectomy (2022) with rubber band ligation (2023), chronic pain syndrome of hips, knees, and hands, on low-dose oxycodone; and an unspecified seizure disorder presented to the hospital for an outpatient colonoscopy. Four months before the hospitalization, she began developing a new persistent aching rectal pain that was initially responsive to acetaminophen and triggered by defecation. She was having three to four loose bowel movements a day, intermittent bright red blood on wiping, and no constipation. Two and a half months before admission, the pain became constant. The rectal pain was worsened by standing or moving, and she was unable to sit on cushioned seats during church services. Two months before admission, a colorectal surgeon diagnosed her with an anal fissure by clinical history, though the anal canal examination was limited without confirmed direct visualization. She was prescribed lidocaine and then nifedipine ointments without pain relief. By this point, the pain was also intermittently radiating to the back of her left thigh, and bowel movements were becoming too painful to tolerate. Two weeks before hospitalization, she had a follow-up appointment where she described worsening of the pain and a plan was made for outpatient botulinum toxin injections to treat anal sphincter muscle spasms and promote perfusion to the area. Given her history of ulcerative colitis, an outpatient colonoscopy was expedited.

On the day of admission, after her colonoscopy, the patient was recovering in the outpatient colonoscopy suite when she suffered a breakthrough seizure, likely provoked by anesthesia, and was admitted to the hospital. After a post-ictal period, she awoke and immediately reported a new burning and more intense rectal pain that was described as “glass shards” in her anal canal. In addition, this pain radiated to her vaginal area, the left lower quadrant of her abdomen, and down her left thigh. Her diarrhea had ceased at this point. The antecedent colonoscopy demonstrated normal-appearing ileum, cecum, and colon. The distal rectum had limited (5 cm), faint, patchy erythema, with biopsies performed, and thought to be non-contributory to her presentation. Histology showed “colonic mucosa with focal acute inflammation and scattered Paneth cells,” which were non-specific for any diagnosis and not compatible with a diagnosis of ulcerative colitis flare or other inflammatory bowel disease. Non-steroidal anti-inflammatory drugs (NSAIDs) were briefly trialed and discontinued for lack of effect and risk of ulcerative colitis flare. High-dose steroids were also tried but discontinued due to a breakthrough seizure episode that was attributed to the high-dose steroids per the Neurology consultation team. Dosing of her home opiates had to be increased, and she was given maximum doses of gabapentin, methocarbamol, and intravenous hydromorphone with limited relief. 

On review of systems, there was no associated nausea, emesis, chest pain, shortness of breath, cough, skin rashes, or dysuria. On physical exam, her vital signs were within normal range, her cardiovascular and respiratory exams were unremarkable, she had a soft abdomen with tenderness to palpation of the left lower quadrant, no oral mucosal ulcerations, and no focal neurologic deficits. On rectal exam, there were no skin lesions between the inner gluteal folds, and the external anal sphincter was visualized without the presence of external hemorrhoids. A digital rectal exam and pudendal nerve pathway palpation were attempted with lidocaine but aborted due to intense pain.

The initial differential diagnosis for this rectal pain included rectal wall tear, rectal wall inflammation from ulcerative colitis or infectious proctitis, rectovaginal fistula, atypical lupus flare, spinal cord impingement, nerve root impingement, atypical presentation of a brain lesion corresponding to the rectal area, and proctalgia fugax. During the next two weeks of the patient’s admission, multiple tests were performed. To evaluate for structural etiologies or hallmarks of malignancy, a CT of the abdomen and pelvis with intravenous contrast demonstrated “no acute findings”, and a pelvis MRI with and without contrast demonstrated “no evidence of rectovaginal fistula”. To investigate the possibility of referred pain from central nervous system pathology, an MRI brain with and without contrast was ordered and showed a “normal brain MRI”. MRI of the complete spine with and without contrast showed “no evidence of cord inflammation (…) minimal degenerative disc disease (…) without substantial canal or foraminal stenosis at any spinal level”. It did not show evidence of pudendal nerve inflammation; however, this is not considered reliable for the exclusion of pudendal neuralgia from the diagnosis. Proctalgia fugax was less favored because of the constant nature of the pain. There were paroxysms of pain that happened with bowel movements, but the pain never went away. In case of an atypical SLE flare, Anti-dsDNA, C3 (121.7 mg/dL. Reference range: 81-157mg/dL), C4 (28.23 mg/dL. Reference range: 13-39 mg/dL), and ESR (10 mm/hr. Reference range: <20 mm/hr) were measured, which were all within normal range. CRP was mildly elevated at 0.88 mg/dL (Reference range: <0.29 mm/hr) though this was thought to be non-specific. Despite the lack of EMG capabilities at our institution, the test is not specific enough to confirm pudendal neuralgia. At the completion of all testing, she had been in the hospital for 20 days without relief of her intense pain, only being able to lay on her side as any pressure on the buttocks aggravated her rectal pain. Out of concern for a possible atypical proctitis, she was initiated on a seven-day course of ceftriaxone and metronidazole, without pain improvement.

With multiple negative tests for this constant pain, the diagnosis of pudendal neuralgia was considered. The Nantes criteria were reviewed [3], and the patient met all of them but had not yet tried a pudendal nerve block. The patient eventually received a CT-guided pudendal nerve block with bupivacaine 0.5% and triamcinolone acetonide 40 mg/mL on her thirty-fifth day of hospitalization. After the procedure, her rectal pain was resolved, and she was finally able to sit normally. Post-procedurally, she did have lower extremity numbness with a prolonged time for sensation to return, which was thought to be secondary to incidental sciatic nerve involvement with the block.

## Discussion

Traditionally, pudendal neuralgia is a difficult diagnosis due to a lack of sensitive physical exam maneuvers and radiography [3]. Unfortunately, many patients experience prolonged uncomfortable symptoms before their diagnosis is formalized. In this case, the patient had waited more than a month in the hospital with escalating doses of opiates, costing an estimated $2,800 per day [4].

The inciting event for pudendal neuralgia typically remains unclear though childbirth, chronic constipation, prolonged sitting or hip flexion, and trauma have been implicated as etiologies [1]. Occasionally, repeated microtrauma or bone remodeling can also cause a subacute advancement of pain [1]. In the case of this patient, the initial cause for her rectal pain four months prior to admission was likely due to microtrauma. However, the underlying process was likely exacerbated by pelvic positioning during her colonoscopy, which she only felt once out of her post-ictal state.

Given the intractable pain, the impact on daily activities, and the lack of diagnostic tests/imaging, pudendal neuralgia remains a clinical diagnosis and therefore should be considered in the differential for patients with rectal pain refractory to treatment. After structural and metabolic problems have been ruled out, a pudendal nerve block should be trialed as both a diagnostic and therapeutic modality for the diagnosis of pudendal neuralgia [3,5,6]. Although pharmacotherapy and pelvic physical therapy are first-line in treating this condition, new therapeutic modalities are emerging, including CT-guided pulse-dose radiofrequency of the pudendal nerve, spinal cord stimulation of the conus medullaris, and surgical decompression [6].

In this case, there were a few confounders that prevented timely diagnosis. First, this patient had a complex history of bleeding hemorrhoids and fissures in the two years before admission; however, she had no rectal pain issues prior to this episode that began four months prior to this admission.

Second, the patient was misdiagnosed with a rectal fissure, causing anchoring bias with therapies being directed at treating this condition, despite no evidence of it on colonoscopy. Third, there was the issue of bright red blood on wiping stool, though it ultimately resolved without intervention and the colonoscopy showed no explanatory findings. Fourth, her diagnosis of chronic pain may have led to confirmation bias by thinking that she may have opiate-induced allodynia though she only used low-dose oxycodone sparingly to help with her chronic pain from SLE post-inflammatory arthralgias.

After 35 days of waiting for a diagnosis and effective treatment, the patient’s constant pain was relieved in one afternoon after one specifically targeted intervention: the pudendal nerve block. Although the patient was elated at the relief, it was unfortunate that she had to experience persistent discomfort due to the delayed diagnosis. Per the literature, pudendal nerve blocks vary in efficacy. Approximately, 25% of patients report pain relief for one month or more [1]. In this case, per documentation from outpatient follow-up with Physical Medicine and Rehabilitation specialists, her pain began to recur around nine weeks after the nerve block with subsequent outpatient coordination for a repeat nerve block. As a result, at the end of her hospitalization, she was deconditioned from a month of decreased mobility due to rectal pain and was recommended for placement in an acute rehabilitation facility, where she will continue her care.

## Conclusions

Pudendal neuralgia is an underdiagnosed condition that commonly leads to inadequate pain management for long periods of time before it is clinically diagnosed. By proactively incorporating it into the initial differential diagnosis of refractory rectal pain, physicians can shorten the diagnosis time and patient pain.
